# Neutrophil depletion enhances the therapeutic effect of PD-1 antibody on glioma

**DOI:** 10.18632/aging.103428

**Published:** 2020-08-04

**Authors:** Peng-Fei Wang, Yu-Xiang Zhang, Jing Su, Kun Yao, Shou-Wei Li, Guang-Rui Huang, Chang-Xiang Yan

**Affiliations:** 1 Department of Neurosurgery, Sanbo Brain Hospital, Capital Medical University, Beijing, China; 2 School of Life Sciences, Beijing University of Chinese Medicine, Beijing, China; 3 Department of Pathology, Sanbo Brain Hospital, Capital Medical University, Beijing, China

**Keywords:** gliomas, PD-1, immunotherapy, tumor infiltrating neutrophils

## Abstract

Tumor-infiltrating neutrophils (TINs), the predominant leukocytes in the tumor microenvironment, are important for cancer-related immunosuppression. Combinations of multiple immune checkpoint inhibitors can significantly improve outcomes in murine glioma models. Here, we investigated TIN levels in human glioma samples and tested the antitumor efficacy of neutrophil depletion alone or in combination with an anti-programmed death 1 (PD-1) antibody. To investigate the clinical relevance, we determined the correlation between tumor grade or survival and TIN levels in 202 resected glioma specimens. TCGA and CGGA data were used to validate the results and analyze the biological functions of TINs in gliomas. An orthotopic xenograft glioma mouse model was used to study the therapeutic effect of anti-PD-1 and/or anti-ly6G. Decreased TIN levels correlated with lower grades, mutant isocitrate dehydrogenase, and favorable prognosis, which was validated by CGGA and TCGA dataset results. Bioinformatics analysis revealed that TINs are mainly involved in angiogenic, inflammatory, and interferon-γ responses in gliomas. TINs were positively correlated with programmed death ligand-1 expression. In xenograft models, combined anti-PD-1 and neutrophil depletion therapy significantly inhibited tumor growth and promoted survival. This study demonstrates that TINs were related to glioma tumorigenesis. Targeting neutrophils could thus enhance the therapeutic effect of PD-1 blockade for gliomas.

## INTRODUCTION

Glioblastomas (GBMs), with an incidence of 3.22 per 100,000 individuals, comprise the most common malignant central nervous system tumor [1]. Therapy for these brain cancers requires a combination of surgery, radiotherapy, and chemotherapy; however, no progress has been made in years with respect to these modalities [2]. In the last decade, immune checkpoint inhibitors (ICIs) targeting cytotoxic T-lymphocyte antigen-4 (CTLA4) and programmed cell death protein-1 (PD-1) or its ligands have yielded promising results for several malignancies [3]. However, immunotherapy based on such compounds has shown limited therapeutic efficacy for patients with GBMs [4, 5]. Preclinical studies indicated that a combination of radiotherapy or other ICIs could boost glioblastoma therapeutic outcomes with PD-1 inhibitors [6, 7].

Neutrophils abundantly infiltrate gliomas and are the main constituent of the brain tumor microenvironment (TME) [8, 9]. These cells are also correlated with poor survival as they exert pro-tumor effects by promoting angiogenesis, immunosuppression, and tumor cell infiltration and proliferation in gliomas [10]. Moreover, tumor infiltrating neutrophils (TINs) are immunosuppressive and tightly associated with the mesenchymal subtype of gliomas [11]. In the TME, TINs can suppress T-cells and NK cells [12, 13], which are the main effector cells targeted during cancer immunotherapy. Moreover, neutrophil gene signatures are abundantly present in clinical GBM samples resistant to anti-PD-1 immunotherapy [14]. Thus, it is rational to hypothesize that TINs might facilitate resistance to immunotherapy in gliomas.

Here, we detected TIN infiltration in glioma surgical specimens and explored its clinical significance, validating these clinical results with those derived from TCGA samples. The immunosuppressive mechanism of TINs was further investigated, especially with respect to their relationship with immune checkpoint expression. Lastly, we examined combined therapy targeting PD-1 and neutrophils in an orthotropic murine GBM model.

## RESULTS

### Association between TIN levels and glioma grade and IDH mutation status

The baseline clinical characteristics of the patients are shown in Supplementary Table 1. In the Sanbo cohort, the mean age was 47.11 ± 14.91 years, and 92 (45.5%) patients were females. There were 150 cases diagnosed as GBMs and IDH-1 R132H mutations were found in 27.2%. Radiotherapy and chemotherapy were performed for 123 and 126 patients, respectively.

**Figure 1:**
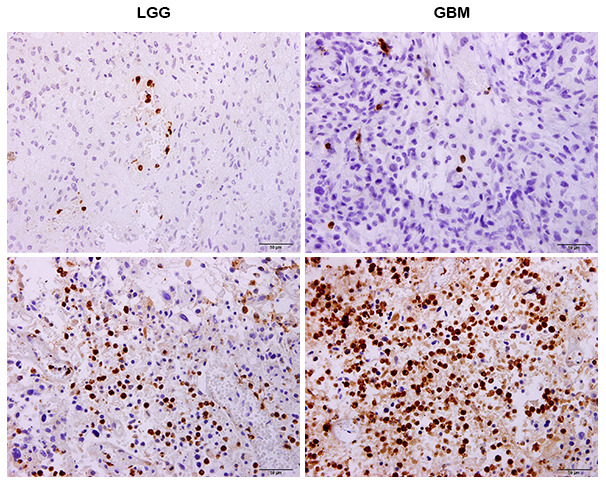
**Immunohistochemistry (IHC) showing that neutrophils differently infiltrate tumors in both LGG and glioblastomas (GBMs).**

**Figure 2:**
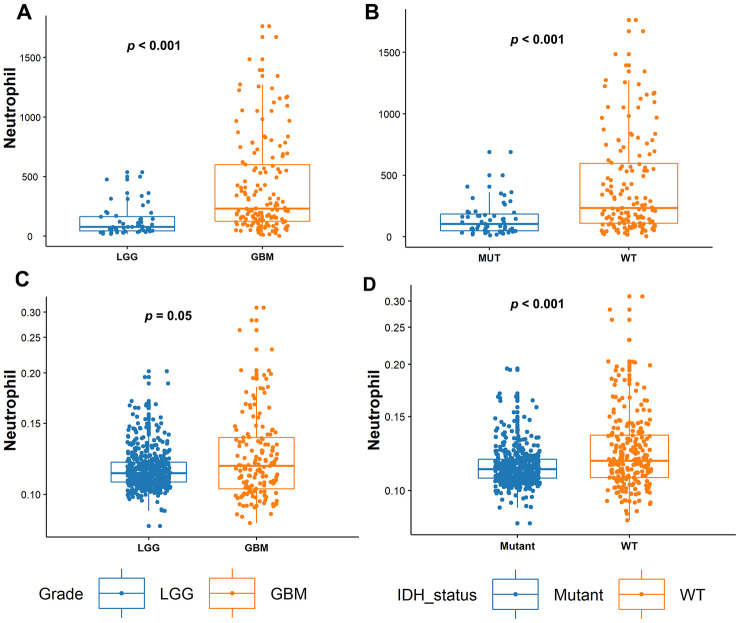
**Tumor-infiltrating neutrophil (TIN) levels increase with tumor grades and in IDH-WT gliomas.** (**A**) In the Sanbo cohort, TIN levels were compared between LGG (n = 52) and glioblastoma (GBM; n = 150) gliomas, and (**B**) in IDH-mutant (Mut; n = 55) and IDH wild-type (WT; n = 147) gliomas. (**C**) In the TCGA dataset, TIN levels were compared between LGG (n = 524) and GBMs (n = 153), and (**D**) IDH-Mut (n = 424) and IDH-WT (n = 234) gliomas.

Neutrophils differentially infiltrated into glioma samples to a great extent (Figure 1). The median number of neutrophils was 183 cells/sample and this ranged from 1~1763 cells/sample. We found that TIN levels were significantly increased in GBMs compared to those in LGGs (p < 0.001, Figure 2A). Next, we compared TINs based on IDH mutation status, as this classifies gliomas based on different genetic changes and survival [15]. It was shown that TINs were dramatically decreased in gliomas samples harboring IDH-1 mutations (p < 0.001, Figure 2B). These results were all validated based on results of the TCGA cohort (Figure 2C and 2D).

### Survival analysis based on TINs in glioma

**Figure 3:**
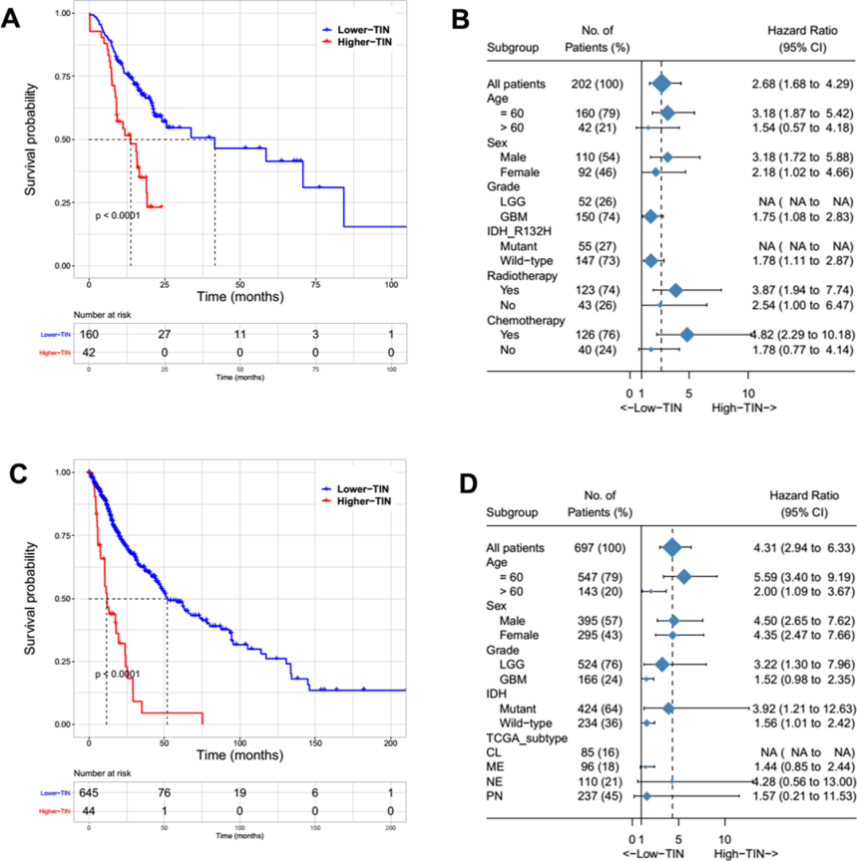
Survival and subgroup analysis of the tumor-infiltrating neutrophils (TINs) to predict overall survival (OS) in patients with gliomas from the Sanbo cohort (**A**, **B**) and TCGA dataset (**C**, **D**).

The patients were first divided into two groups according to TIN levels and the cut-off that could best predict overall survival (OS). The group with higher TIN levels was significantly associated with poor clinical outcomes both in the Sanbo cohort (p < 0.001, Figure 3A) and TCGA cohort (p < 0.001, Figure 3C). Based on subgroup analysis, TINs still predicted OS regardless of IDH mutation status and sex in both cohorts (Figure 3B, 3D). We also found that high TIN levels correlated with poor clinical outcomes in our cohort, but it did not reach statistical significance in the TCGA cohort (Figure 3B, 3D). Whereas previous studies have shown a close relationship between TINs and the mesenchymal subtype [11], this did not predict survival in the TCGA subtype (Figure 3D).

### Biological significance of TINs in gliomas

**Figure 4:**
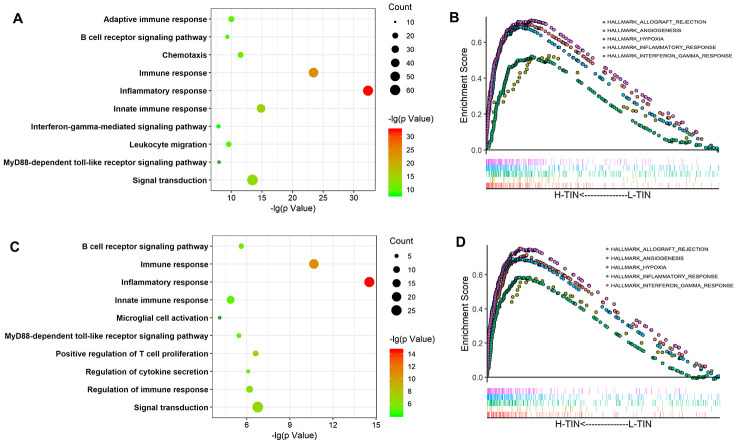
**Biological process of tumor-infiltrating neutrophils (TINs) in gliomas.** GO analysis of the TCGA and CGGA datasets shows that TINs are involved in the inflammatory response, immune response, and other GO immune functions (**A**, **C**). GSEA analysis showed that TINs participate in the inflammatory response, angiogenesis, and interferon-γ responses (**B**, **D**).

To explore the main biological processes associated with TINs in glioma, Spearman correlation analysis (|r| > 0.5 and p< 0.05) was conducted to identify related genes. In total, 373 and 141 genes were selected as being closely associated with TIN levels based on TCGA and CGGA datasets, respectively. Moreover, GO analysis was performed at the DAVID website to explore biological processes associated with TINs in gliomas. Related genes were mainly involved in immune and inflammatory responses (Figure 4A, 4C). Furthermore, gene set enrichment analysis (GSEA) analysis confirmed that TINs mainly participated in the inflammatory response, interferon-γ response, angiogenesis, and response to hypoxia in both the TCGA and CGGA datasets (Figure 4B, 4D).

### Co-expression of PD-L1 with TINs

**Figure 5:**
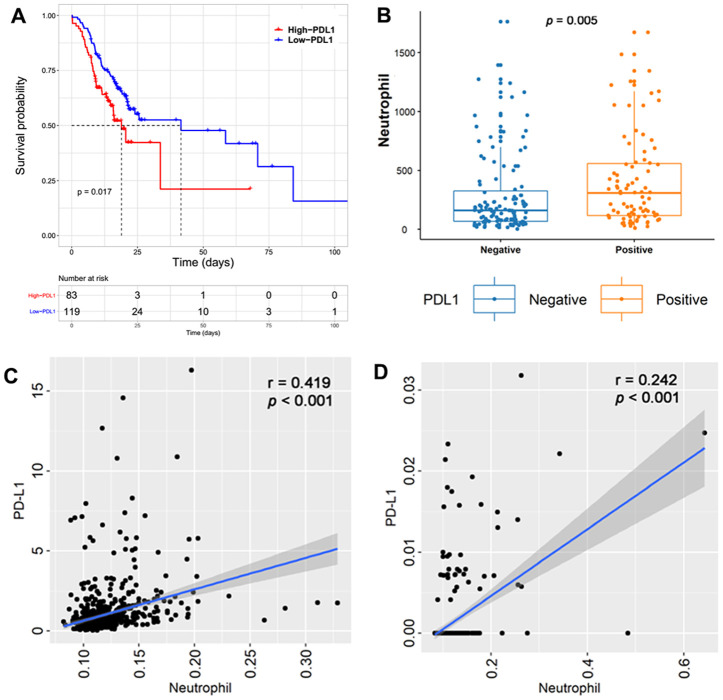
Tumors with higher PD-L1 expression were associated with a poor clinical outcome in the Sanbo cohort (p =0.017, (**A**). Tumor-infiltrating neutrophils (TINs) were significantly increased in tumors with PD-L1 positivity (p = 0.005, (**B**). Spearman correlation between TINs and *PD-L1* mRNA levels showed that *PD-L1* transcript levels were significantly positive with TINs in TCGA (p < 0.001, (**C**) and CGGA cohorts (p < 0.001, (**D**).

Immunohistochemistry (IHC) was performed to detect PD-L1 expression in glioma samples The representative immunohistochemical images for PD-L1 are shown in Supplementary Figure 1. This showed that tumors with higher PD-L1 expression were associated with poor clinical outcomes (p = 0.017, Figure 5A). Moreover, TINs were significantly increased in PD-L1-positive tumors (p = 0.005, Figure 5B). Next, we performed Spearman correlation analysis, comparing TINs with *PD-L1* mRNA levels. This showed that *PD-L1* transcript levels were significantly and positively associated with TINs in TCGA (Figure 5C) and CGGA cohorts (Figure 5D).

### Therapeutic effect of targeting TINs and PD-1 based on an intracranial xenograft mouse model

**Figure 6:**
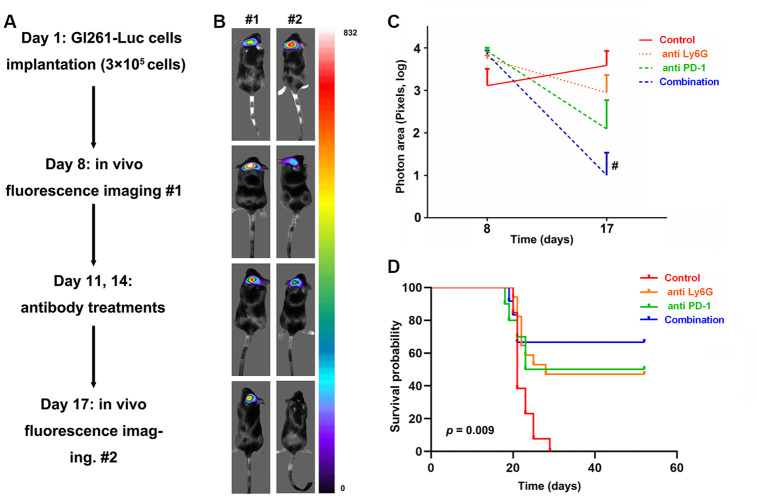
**In vivo evaluation of targeting tumor-infiltrating neutrophils (TINs) and PD-L1 to treat glioblastomas (GBMs) in mice.** The study flow of animal experiments (**A**). The representative image of tumor changes before and after antibody treatments (**B**). The tumor sizes significantly decreased with the dual therapy comprising anti- PD-1 and depletion of neutrophils compared to those in the control groups (**C**). Survival analysis showed that the mice receiving dual therapy had the longest survival times (**D**).

A flow diagram of this experiment is presented in Figure 6A. Briefly, the intracranial xenograft mouse model was generated on day 1 and fluorescence imaging was performed on day 8 to exclude mice that failed to develop gliomas. Finally, 52 mice were included in our study and were randomly divided into four groups as follows: control (n = 13), anti-PD-1 antibody (n = 10), anti-Ly6G (n = 17), combined therapy (n = 12). On the 11^th^ and 14^th^ days, each group received antibody treatments. On day 17, fluorescence imaging was performed to evaluate changes in tumor size.

A representative image of tumor sizes is presented in Figure 6B. There were no differences in tumor sizes among the four groups (p > 0.05, Figure 6C). After treatment, mice receiving dual therapy comprised of an anti-PD-1 antibody and neutrophil depletion showed a significant decrease in tumor size compared to that in control mice (p = 0.049, Figure 6C). Moreover, survival analysis was performed to evaluate the therapeutic effect. No mice in the control group exhibited long-term survival. However, mice administered combined treatment had the longest survival times (p = 0.009, Figure 6D). In addition, for eight of the 12 mice, tumors completely regressed.

## DISCUSSION

In this study, we characterized TINs in glioma based on clinicopathological factors. TINs were increased in higher-grade tumors and negatively affected patient survival by mediating an inflammatory response, suppressing the immune response, and promoting angiogenesis. Moreover, the presence of TINs was associated with PD-L1 expression, suggesting the utility of a combined treatment strategy for these two targets. Accordingly, PD-1 inhibitors and neutrophil depletion significantly improved survival outcomes in a xenograft mouse model of intracranial glioma. Our study thus suggests that TINs strongly suppress the effect of PD-1 inhibitors in glioma.

Circulating neutrophils or elevated neutrophil-to-lymphocyte ratios were previously found to be associated with poor clinical prognosis [16, 17]. Although higher levels of TINs predict favorable clinical outcomes for gastrointestinal tumors [18, 19] or lung cancers [20], we first observed that TINs negatively influence the survival of patients with gliomas. Previously, it was shown that tumor-associated neutrophils can release S100A4, which increases tumor proliferation and the transformation to a mesenchymal subtype in gliomas [21]. Moreover, TINs induce angiogenesis and immunosuppression in gliomas [5, 10]. In addition to these established functions, our GSEA analysis suggested that TINs are associated with the enrichment of gene sets involved in interferon-γ signaling, which is a strong predictive marker of immune checkpoint inhibitor success in cancer [22, 23]. Moreover, PD-L1 was positively expressed with TINs, and these results prompted us to treat gliomas by targeting neutrophils and PD-L1. The depletion of neutrophils was previously found to prolong survival in glioma mouse models [24]. However, our results showed that simultaneous anti-neutrophil and PD-1 inhibitor treatment was required to induce disease regression in most mice with glioma. These results imply a novel strategy to enhance the efficacy of PD-1 inhibitors for the treatment of gliomas. However, one of the shortcomings of our study is that we did not definitively demonstrate causality. Future studies were needed to establish this and to determine if other immune cells are affected.

Glioma-associated neutrophils were found to be associated with resistance to PD-1 inhibitors in our study and previously to anti-VEGF therapy [25]. Moreover, the depletion of neutrophils can improve survival outcomes for mice with glioma [24]. However, it is difficult to deplete patients of neutrophils as they play a pivotal role in responses to infections. Restricting neutrophil-associated chemokines is one therapeutic strategy; further, a variety of chemokines attract neutrophils in cancer [26]. Moreover, there are numerous intrinsic genetic changes or oncogenic signaling pathways associated with gliomas that could be related to neutrophil chemotaxis in this disease, as such changes were previously found to be related to immune suppression [14, 27]. Thus, future studies should be conducted to investigate the association between TINs and genetic changes in gliomas.

## CONCLUSIONS

In conclusion, our results provided translational evidence suggesting that TINs might serve as a candidate target to increase the sensitivity of gliomas to PD-1 antibody. A combination of PD-1 blockade and neutrophil depletion was also found to exert significant therapeutic effects in an in vivo glioma model. Future investigation is encouraged to target TINs in gliomas to alleviate their negative effects on PD-1 inhibitors.

## MATERIALS AND METHODS

### Patients and specimens

We retrospectively reviewed 202 cases of patients who underwent surgery at Sanbo Brain Hospital from 2016 to 2018. The patients followed an adjuvant regimen according to Stupp’s protocols [2]. All patients were diagnosed with gliomas according to histopathology [28]. We calculated the OS in months and defined it as the period from operation to death or censored. Our follow-up ended in April 2019. The ethics committee of Sanbo Brain Hospital and Beijing University of Chinese Medicine approved all procedures based on patients and animals, respectively.

### Bioinformatics

The TCGA transcriptome data were downloaded from the GDC Data Portal (https://portal.gdc.cancer.gov/). TIN infiltration based on CGGA and TCGA data was calculated using the Timer website (https://cistrome.shinyapps.io/timer/) [29]. Genes significantly related to TINs were identified based on Spearman correlations. Further, the gene ontology (GO) of the related genes was analyzed using the DAVID website (http://david.ncifcrf.gov/) [30]. GSEA was used to investigate the potential role of TINs using “hallmark gene sets (h.all.v7.0.symbols)” with Java 4.0 Desktop Application (http://software.broadinstitute.org/gsea/index.jsp) [31].

### IHC

Immunohistochemical staining was performed on formalin-fixed, paraffin-embedded surgical specimens of tumors, as described in our previous study [32]. Primary antibodies specific for MPO (ZSGB-BIO, Beijing, China), PD-L1 (Cell Signaling Technology, Danvers, USA), and IDH-1^R132H^ (Dianova, Hamburg, Germany) were used. The number of neutrophils was recorded as the total number based on the three most obvious regions. Two experienced observers (K Yao & YX Zhang) who were blinded to the clinical data evaluated immunostaining.

### Animal experiments

The intracranial xenograft mouse model was generated according to our previous report [33]. Six-week-old female C57/BL mice were purchased from Beijing Vital River Laboratory Animal Technology Co., Ltd. (Beijing, China) and bred in rooms under controlled conditions of temperature, humidity, photoperiods, and air exchange. GL261-Luc cells were cultured for up to three passages and maintained in logarithmic growth phase. Then, the mice were anesthetized (1% pentobarbital, 0.4 mL/100 g mouse body weight, i.p.) and approximately 3 × 10^5^ GL261-Luc cells, resuspended in 5 μL of PBS, were injected stereotactically into the left striatum. Animals were euthanized when they demonstrated signs of morbidity including hunched posture, lethargy, difficulty moving, and weight loss.

### Therapeutic antibodies

An anti-PD-1 antibody (200 μg per mouse, clone 29F.1A12; Bioxcell) was injected intraperitoneally into animals. To deplete neutrophils, an anti-Ly6G antibody (200 μg per mouse, clone 1A8; Bioxcell) was also injected intraperitoneally. The TINs were totally depleted by anti-Ly6G antibody (Supplementary Figure 2). A matched isotype rat IgG2A antibody (BE0089; Bioxcell) served as a control.

### In vivo fluorescence imaging

In vivo fluorescence imaging was performed on days 8 and 17 after intracranial implantation. For this, mice were injected with luciferin (100 μg/g) in PBS; after 10 min, the mice were anesthetized using isoflurane gas and imaging and measurements were performed using Metamorph software (Molecular Devices, Silicon, USA); tumor areas (pixels) were calculated to evaluate changes in tumor size.

### Statistical analysis

R (3.5.2) language, SPSS 22.0, and GraphPad 6.0 were the main tools used to analyze data and generate figures. Differences in TIN infiltration based on tumor grade, IDH mutations, and PD-L1 expression were compared using the Wilcoxon test. The correlation between TIN levels and gene expression was assessed by Spearman analysis. The cut-off value for TIN to predict survival was determined using X-tile 3.6.1 [34]. Differences in tumor sizes between groups of mice were assessed by an analysis of variance (one-way ANOVA) and confirmed by an LSD test. P < 0.05 was considered statistically significant.

## Supplementary Material

Supplementary Figures

Supplementary Table 1
