# Supplementary material for: Neutrophil depletion enhances the therapeutic effect of PD-1 antibody on glioma
Source: Aging (Albany NY). 2020 Aug 4;12(15):15290–301. doi: 10.18632/aging.103428 (PMC7467393; doi:10.18632/aging.103428)
Supplement: Supplementary Figures [file aging-12-103428-s002..pdf]

SUPPLEMENTARY FIGURES

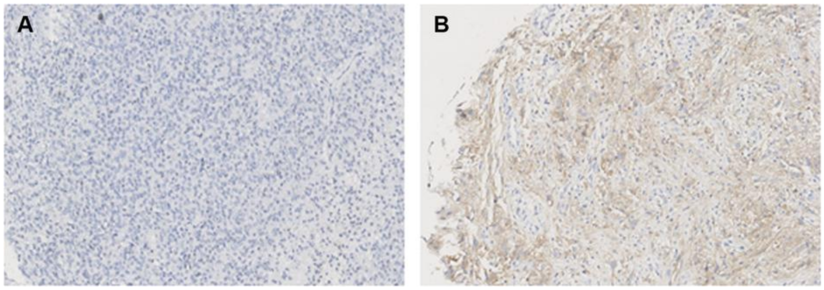

Supplementary Figure 1. Representative image of PD-L1 expression in gliomas. (**A**) Negative membrane staining for PD-L1. (**B**) Positive membrane staining for PD-L1.

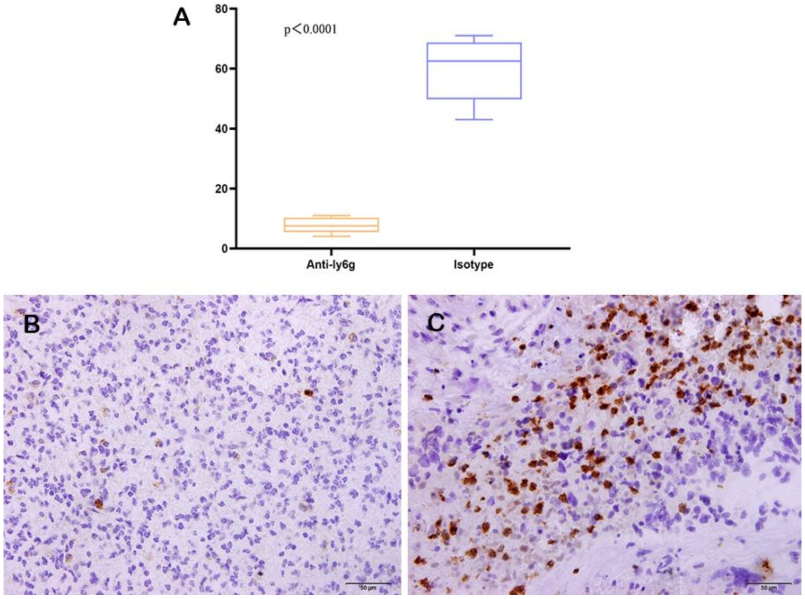

Supplementary Figure 2 The experiment of neutrophil depletion. There were six mice treated with. TINs were significantly deceased in the group treated with anti-Ly6G antibody (**A**). The representative image of IHC from six mice, which were treated with anti-Ly6G antibody (**B**) or matched isotype (**C**).
