# Supplementary material for: Neutrophil depletion enhances the therapeutic effect of PD-1 antibody on glioma
Source: Aging (Albany NY). 2020 Aug 4;12(15):15290–301. doi: 10.18632/aging.103428 (PMC7467393; doi:10.18632/aging.103428)
Supplement: Supplementary Table 1 [file aging-12-103428-s001..pdf]

SUPPLEMENTARY TABLE

Supplementary Table 1. Baseline characteristics.

**Age**	
Mean ± SD	47.11 ± 14.91
Median (range)	47 (10-86)
**Gender**	
Female	92 (45.5%)
Male	110 (54.5%)
**Tumor grade**	
LGG	52 (25.7%)
GBM	150 (74.3%)
**IDH-1^{R132H}**	
Mutation	55 (27.2%)
Wild type	147 (72.8%)
**TIN (No./Sample)**	
Median (range)	183 (2-1763)
**Radiotherapy**	
Yes	123 (74.1%)
No	43 (25.9%)
**Chemotherapy**	
Yes	126 (75.9%)
No	40 (24.1%)
